# Is Limited English Proficiency or Speaking Spanish Associated with Medicare Advantage Enrollment?

**DOI:** 10.1093/geroni/igaf122.264

**Published:** 2025-12-31

**Authors:** Lilly Estenson, Eric Roberts, Mireille Jacobson

**Affiliations:** University of Southern California, Los Angeles, California, United States; University of Pennsylvania, Philadelphia, Pennsylvania, United States; University of Southern California, Los Angeles, California, United States

## Abstract

Medicare beneficiaries increasingly choose to receive their Medicare benefits through private Medicare Advantage (MA) plans. While prior research has identified higher MA enrollment rates among Latino, Black, and low-income beneficiaries, trends among beneficiaries who speak Spanish and/or have limited English proficiency (LEP) are less clear. Accordingly, we used nationally representative Medicare Current Beneficiary Survey data and linear probability models to estimate differences in MA enrollment by Spanish language and LEP status. We adjusted for social, health, and local MA market characteristics, year, and county fixed effects. Among approximately 30,000 respondents, 6.0% spoke Spanish, and separately, 6.1% had LEP. Spanish-speaking beneficiaries had substantially higher rates of MA enrollment (58.9%) compared to non-Spanish-speaking beneficiaries (36.2%). Among Spanish speakers, beneficiaries with LEP had higher MA enrollment than beneficiaries with English proficiency (60.9% vs 51.1%). Among non-Spanish speakers, differences in MA enrollment by LEP status were minimal (36.6% vs 36.3%). In adjusted models, neither speaking Spanish nor LEP status was associated with MA enrollment when examined in the full sample. LEP status was not associated with MA enrollment when examined among Spanish- and non-Spanish speakers separately. Our results highlight higher rates of MA enrollment among Spanish-speaking beneficiaries, especially Spanish speakers with LEP. However, after adjusting for covariates, neither speaking Spanish nor LEP appear to independently account for MA enrollment differences. Additionally, our results support prior evidence of racial/ethnic and socioeconomic MA enrollment differences. Understanding the role of social factors in choosing MA can inform MA marketing, benefit design, and quality improvement policies.

